# Presynaptic Inhibition in the Striatum of the Basal Ganglia Improves Pattern Classification and Thus Promotes Superior Goal Selection

**DOI:** 10.3389/fnsys.2015.00152

**Published:** 2015-12-15

**Authors:** David J. Schwab, James C. Houk

**Affiliations:** ^1^Department of Physics and Astronomy, Northwestern UniversityEvanston, IL, USA; ^2^Department of Physiology, Northwestern UniversityChicago, IL, USA

**Keywords:** presynaptic inhibition, striatum, basal ganglia, schizophrenia, pattern classification

## Abstract

This review article takes a multidisciplinary approach to understand how presynaptic inhibition in the striatum of the basal ganglia (BG) contributes to pattern classification and the selection of goals that control behavior. It is a difficult problem both because it is multidimensional and because it is has complex system dynamics. We focus on the striatum because, as the main site for input to the BG, it gets to decide what goals are important to consider.

The basal ganglia (BG) select coordinated sets of behavioral goals in parallel loops to and from different areas of the cerebral cortex. The main input stage in these BG loops is the striatum and cortical neurons send numerous excitatory axons to the various cell types contained within the caudate, putamen, and ventral divisions of the striatum. The main flow of information is direct from cortex to spiny projection neurons (SPNs) out to one of the next stages of processing in the different BG loops and finally back to the area of cortex that generated the main excitatory input, thus forming a closed loop. Each SPN emits a prominent array of inhibitory collaterals before its axon leaves the striatum. The SPNs constitute 95% of the neurons within the striatum, and they are all GABAergic, although SPNs also contain an opiate as a co-transmitter. There are various types of interneuron within the striatum, many of which are also GABAergic (Tepper et al., 2010). The neurons of the striatum are prominently innervated by the dopamine (DA) neurons that signal the likelihood of future reward and produce both short and long-term neuromodulatory responses (Gruber et al., 2003).

This review attempts to provide a new perspective on the computational functioning of the striatum. The primary function we will focus on is goal selection. We would ultimately like to understand the computations the BG performs, so it's important to consider the structure and information content of the inputs to the striatum. The spiny neurons in the striatum receive input from the cortex, with approximately 380,000 axons innervating the dendritic tree of each spiny neuron. Notably, neighboring striatal neurons with overlapping dendritic trees are believed to share very few upstream cortical neurons (Kincaid et al., 1998). Moreover, cortical neurons whose axons overlap are believed to nonetheless share few spiny neuron targets. As a result, spiny neurons located near each other spatially encode relatively independent components of cortical information.

Notably, however, the spiking activity of the spiny neurons is quite sparse. Given the significant level of glutamatergic excitation arising from the cortex, inhibition must therefore be playing a significant role in stabilizing the activity level in the striatum. It is well-known that there exist both feedforward as well as feedback forms of inhibition at work in the striatum, but it is unclear which form, if any, dominates as well as the functional role of each. It is worth noting, however, that feedback inhibition that is activity based provides a competitive interaction that can mediate variants of winner-take-all computations that may be vital for goal selection (Wickens et al., 2007). Feedforward inhibition, while able to sculpt the structure of cortical input, is unable to explicitly execute a competitive computation.

It was argued that, despite the potential computational benefits of feedback inhibition, feedforward inhibition dominates in the striatum (Tepper et al., 2004). Indeed the feedforward interneurons provide strong inhibitory signals whereas feedback inhibition was measured directly between pairs of spiny neurons and was demonstrated to be relatively weak (Koos et al., 2004). This has lead to the hypothesis that feedback-based inhibition is too weak to play much of a role. It is however important to recognize that the total contribution due to feedback inhibition depends not only on the efficacy of a single upstream inhibitory cell but additionally on the number and layout of all inhibitory synapses between spiny neurons (Wickens et al., 2007). These numbers have been estimated very roughly, see Figure 1, and it has been argued that the total level of feedback inhibition is sufficient to provide a strong effect at the network level (Wickens et al., 2007; Ponzi and Wickens, 2013). But these analyses appear to depend critically on many poorly known parameters and it is therefore unclear how significant a role postsynaptic feedback inhibition plays.

**Figure 1:**
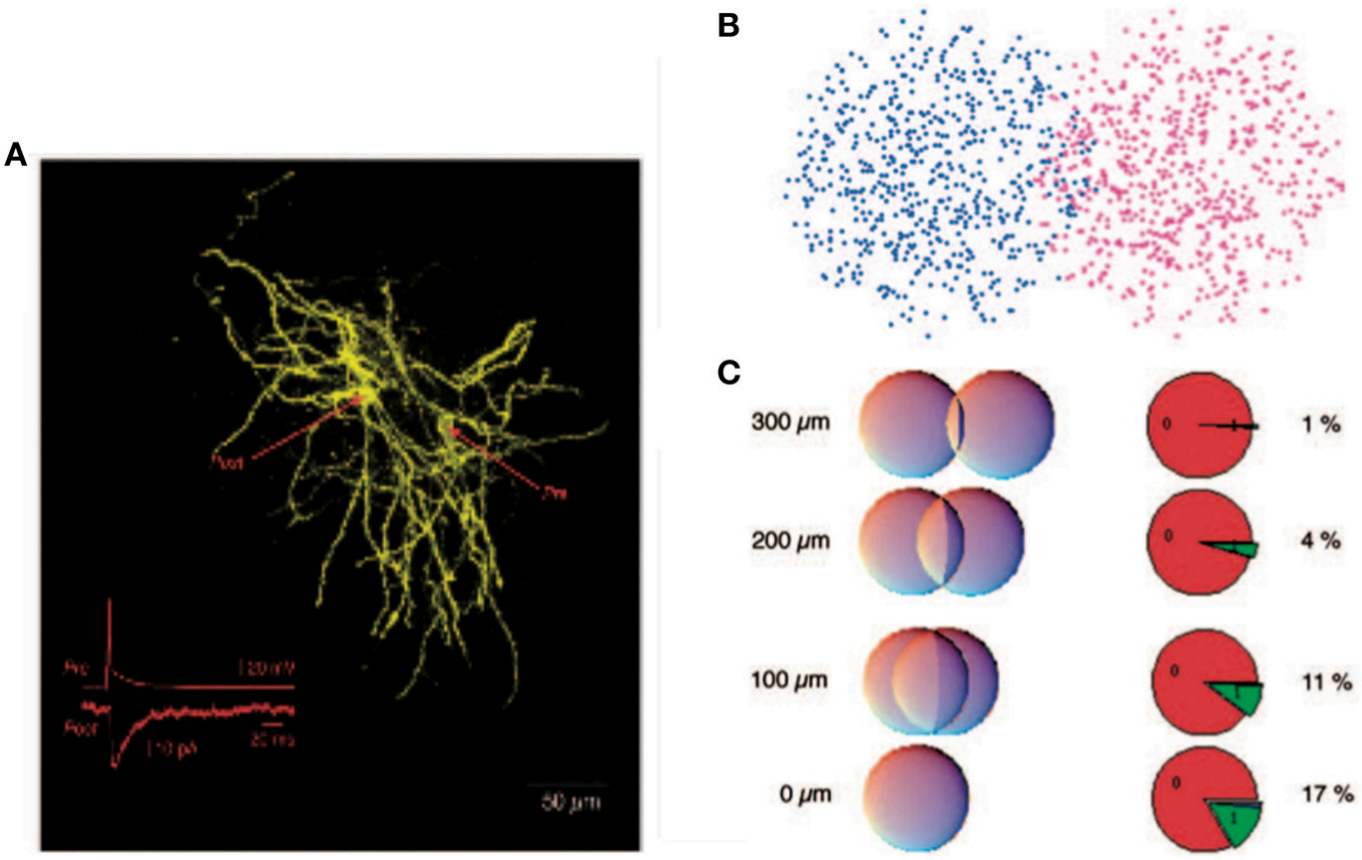
**Reproduced from Wickens et al. (2007), estimate of probability of synaptic contact. (A)** A pair of filled spiny projection neurons showing their overlapping axonal and dendritic trees. Inset shows the response evoked in the postsynaptic cell by firing of the presynaptic cell. **(B)** Model of a pair of cells as “clouds” over overlapping dendritic and axonal sites. **(C)** Results of using a hypergeometric distribution of synapse locations calculate the probability of a connection at distances ranging from 0 to 300 μm between centers of the cells.

The central theme of this review is that a vital component of competitive feedback may be mediated through presynaptic inhibition. Before the report by Houk et al. (2007) the possibility that competitive feedback relies on presynaptic, as opposed to postsynaptic, inhibition had not been considered. This is surprising since presynaptic inhibition of cortical input to the striatum had been demonstrated electrophysiologically (Calabresi et al., 1990) and morphologically (Nisenbaum et al., 1992; Lacey et al., 2005). We argue here that presynaptic inhibition may confer numerous advantages over postsynaptic inhibition, from providing a uniform strength of inhibition independent of the postsynaptic cell's underlying somatic potential to the facilitation of longer time dynamics within the spiny neuron population that may promote reinforcement learning.

It has been argued that feedback inhibition between spiny neurons is weak (Tepper et al., 2004), but the measurements of the efficacy of inhibition between two spiny cells only measured postsynaptic effects, thereby potentially underestimating the strength of feedback in the striatum. Notably, these measurements would miss any presynaptic effects. Additionally, as illustrated in Figure 2 presynaptic inhibition has the advantage of being of uniform strength, irrespective of the somatic potential of the receiving cell (Koch, 1998; Houk et al., 2007). In contrast, postsynaptic inhibition depends on the relationship between the reversal potential of the synapse and the underlying somatic potential, possibly even becoming excitatory when the receiving cell is sufficiently hyperpolarized. Interestingly, however, presynaptic inhibition can act as effectively excitatory or inhibitory but only in conjunction with other inputs. If the presynaptic inhibition is inhibiting an excitatory synapse, it functions as inhibitory. But if the presynaptic inhibition is onto an inhibitory synapse, it effectively acts excitatory due to the relief of inhibition. (Of course related effects also occur at the network level—inhibiting an inhibitory neuron increases the net excitation in the population for both pre- and post-synaptic inhibition.) It is also worth noting that presynaptic inhibition only provides an effect when the synapses it is targeting are active. Otherwise, its effect is silent and hidden. All of these effects change the computational functioning of presynaptic inhibition from its more familiar postsynaptic counterpart.

**Figure 2:**
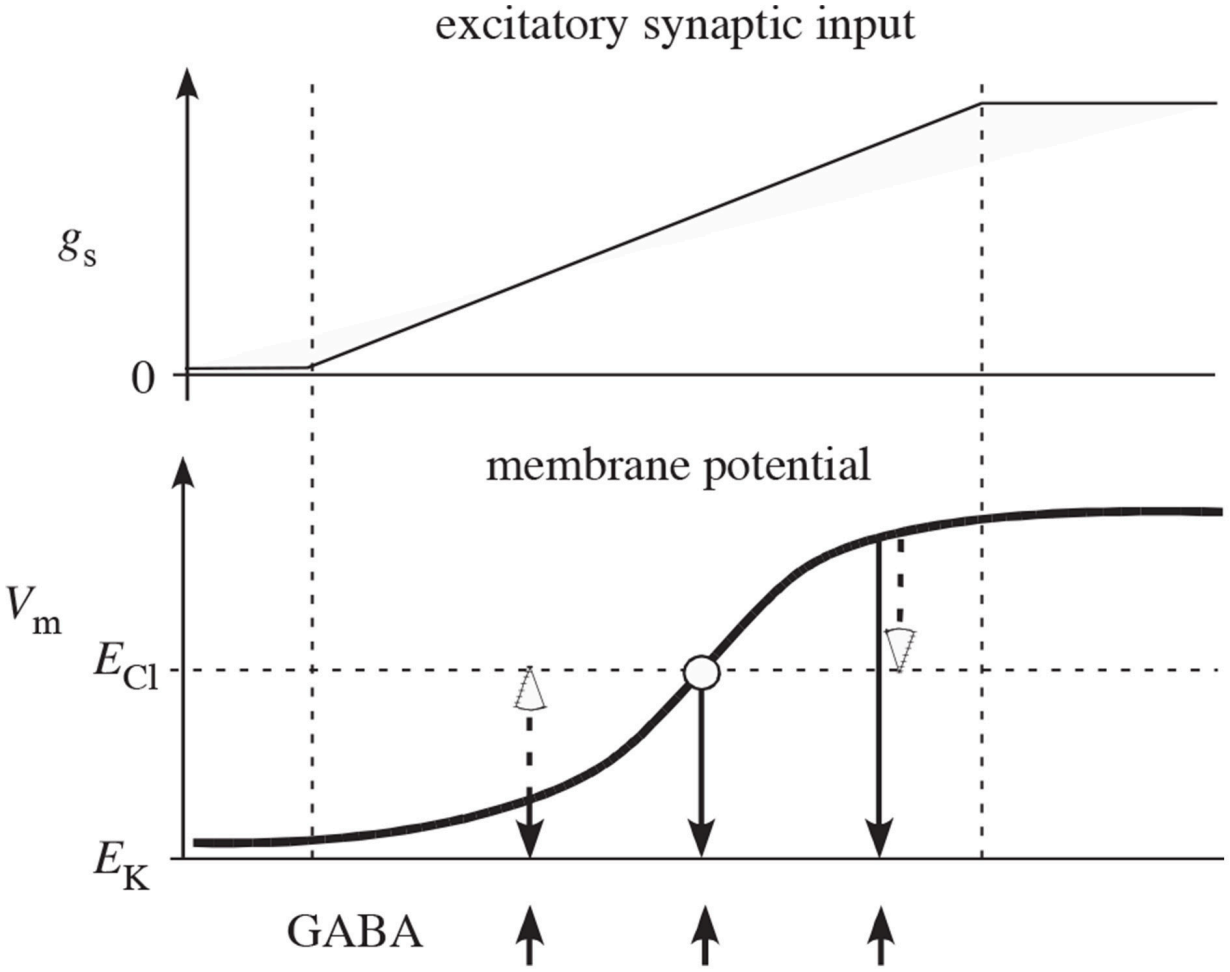
**Reproduced from Houk et al. (2007) schematic illustration of why competition mediated by presynaptic inhibition is more effective than competition mediated by postsynaptic inhibition**. The two plots show net excitatory synaptic input (g_s_) from cortex and membrane potential (V_m_) of a spiny neuron as the cortical input slowly increases (between the two vertical dashed lines). In the absence of synaptic input, V_m_ is near the potassium equilibrium potential E_K_. As synaptic input g_s_ increases, V_m_ moves in the positive direction in a sigmoidal fashion (typical of a down-to-up state transition). The upward arrows indicate times of GABA release from inhibitory collaterals. The open arrows illustrate how postsynaptic inhibition actually depolarizes (excites) spiny neurons that are in the down-state and only mediates shunting inhibition when V_m_ is at the chloride equilibrium potential E_Cl_. The downward closed arrows show that presynaptic inhibition always decreases membrane potential (inhibits) and therefore is qualitatively more effective than postsynaptic inhibition.

We next turn to the physiological substrate that may mediate presynaptic inhibition in the striatum. In particular, recent experimental work by Blomeley and Bracci (2011) indicates that there exists opioid-based presynaptic inhibition between spiny cells. Through paired recordings, they found that a short burst of activity in a spiny neuron depressed the strength of cortical inputs by 17% on average to approximately half of nearby cells. It is important to note that such inhibition is quite nonspecific and requires diffusion through the extracellular medium. As a result, it is clear that this mechanism would not target particular synapses, dendritic branches, or even cells. Rather the effect may be spread throughout a spatial region accessible via diffusion. A natural question is why the spiny neurons make particular, direct inhibitory synapses rather than simply releasing GABA and opioids into the extracellular medium. One possibility is that it is advantageous or even physiologically necessary to anchor the location of modulator release.

The perspective we thus present for competitive inhibition in the striatum is illustrated in Figure 3. In addition to the traditional postsynaptic inhibition mediated by GABAa synapses, presynaptic inhibition of corticostriatal inputs mediated by GABAb and opioid receptors act on slower timescales. Furthermore, there are other substances that evoke the presynaptic calcium transients known to mediate presynaptic inhibition (Lovinger and Choi, 1995; Kupferschmidt and Lovinger, 2015), and they should also be considered as part of the framework. All of these effects in combination enact a competitive dynamics between spiny neurons to yield goal selections.

**Figure 3:**
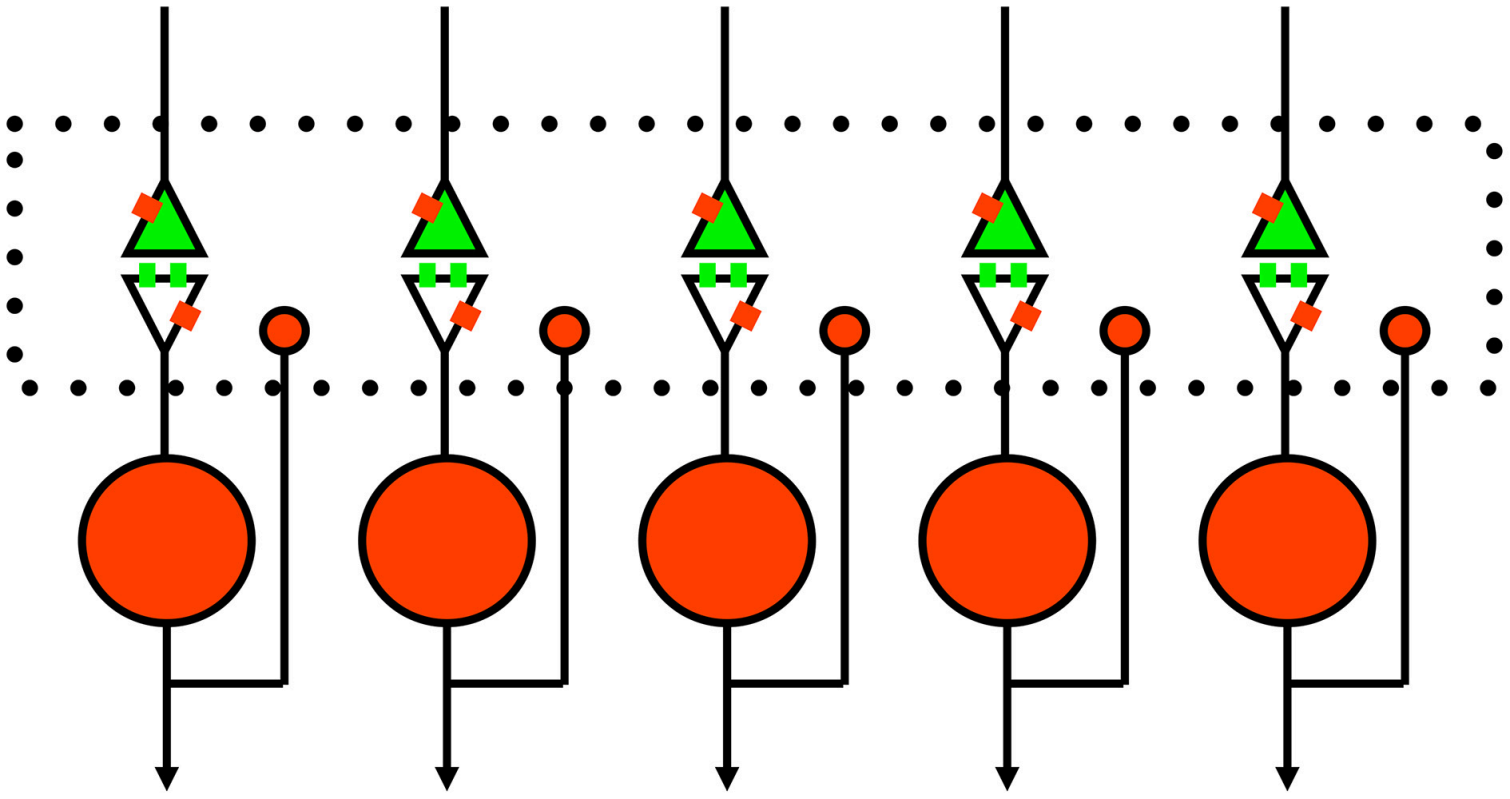
**Schematic of proposed rule of presynaptic inhibition in mediating competition between spiny neurons**. Green, excitatory glutamate input from cortex or thalamus with receptors on spines. Red, inhibitory GABA neurons with single collaterals. Small red rectangles represent GABAb receptors that lack specific synaptic inputs (Lacey et al., 2005). The large dotted rectangle represents extracellular space for diffusion of GABA and opioids to presynaptic and postsynaptic sites; glutamate just diffuses across the narrow synaptic cleft. Of course, this is a simplified 1-dimensional model and the real system (long-axis of dotted box) is actually three-dimensional.

As shown schematically in Figure 4, the effects of presynaptic inhibition persist for a fairly long duration, up to a second, much longer than any of the synaptic time constants in the system. (It is also worth noting that the effect takes a few hundred milliseconds to ramp up to full strength.) By explicitly producing a slow inhibitory dynamics, this may encourage dynamical competition that robustly persists for similarly long times. Additionally, simulations of postsynaptic inhibitory spiny neurons driven by cortical input demonstrate a transition regime that promotes long time dynamics (Ponzi and Wickens, 2013). However, for the system to sit in this special transition regime with slow dynamics, the system parameters must be fine-tuned so that the network is poised precisely in the transition regime. Rough estimates indicate that the striatum may be near this regime, but the requirement for fine-tuning is worrisome. Interestingly, presynaptic inhibition may broaden the range of parameters for which the system is able to support long time dynamics, similar to the proposed role of synaptic depression in enhancing scale-free avalanche distributions in model cortical circuits (Levina et al., 2007). This, of course, must be tested in a computational model.

**Figure 4:**
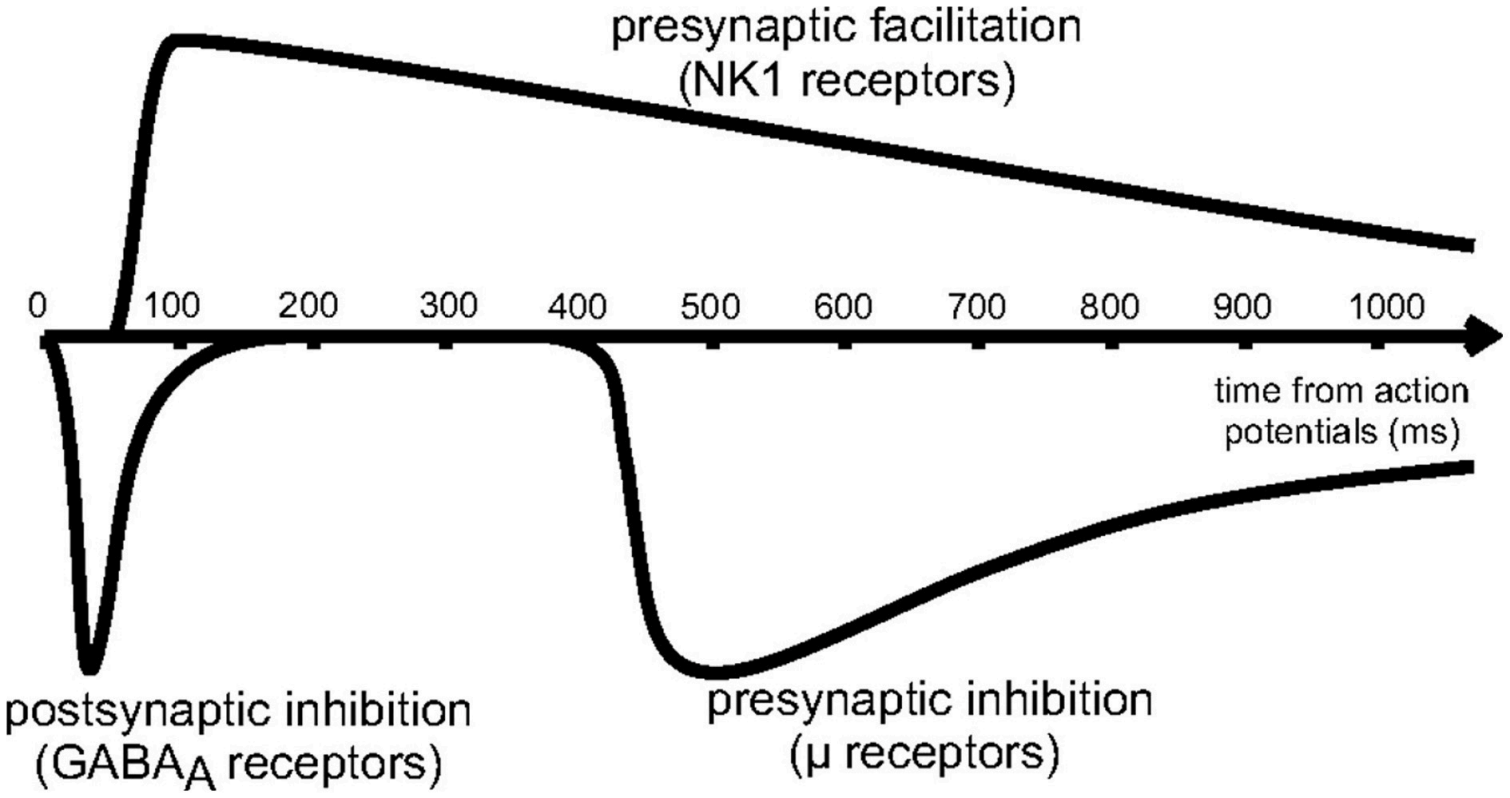
**Reproduced from Blomeley and Bracci (2011)**. Schematic of the time course of inhibitory interactions between spiny neurons. Fast postsynaptic inhibition is mediated by GABAa receptors, while μ-opioid receptors enact presynaptic inhibition on a slower timescale, lasting for approximately 1s.

In the machine learning literature, forms of presynaptic inhibition have been proposed to develop novel algorithms (Yuille and Greynacz, 1989; Spratling, 1999; Spratling and Johnson, 2002). However, the non-specific presynaptic inhibition mediated by opioids differs dramatically from the forms discussed in the machine learning literature. In the latter case, presynaptic inhibition must be able to target individual inputs, thereby providing exquisite control over inhibitory competition, thus leading to greater specificity during learning. However, such precise interactions are not biologically plausible and are unlikely to be employed anywhere in the brain.

Finally, we briefly discuss the consequences of these ideas in the context of Distributed Processing Modules (DPMs) (Houk, 2005), a proposed model of whole-brain learning and control inspired by the anatomical connectivity between the cerebral cortex, the BG, and the cerebellum. In brief, the loops between the BG and cerebral cortex are thought to discover goals, through a reinforcement learning-like process, while the loops between the cerebellum and cerebral cortex generate intentions, such as a movement command, i.e., how to practically enact these goals. An analogy would be that the BG to cortex loop is the Professor that envisions a research project and the cerebellum to cortex loop is the graduate student that gets the job done in practice. Such modules have been proposed to be organized into a hierarchy to learn interrelated goals and varying levels of abstraction. Here we addressed the physiological substrate through which the loop from the BG through cortex selects goals. In particular, we suggested that competition between the SPNs in the striatum is vital to this process and that presynaptic inhibition plays a key role in mediating such a competition. The end result is that, associated to a given experience, a limited number of spiny neurons win and represent the preeminent goal to be enacted by the cerebellar to cortical loop.

To get more specific, we review next a neural network model of serial order processing that was based on the overall function of the cortical-basal ganglia loop (Beiser and Houk, 1998). As summarized in Houk et al. (2007), the focus of the Beiser-Houk model was on serial order processing, a crucial feature of higher order intelligence (Lashley, 1951), The model's ability to encode the serial order of events resulted from the combination of three computational features:

1. Pattern classification of a cortical input vector by computations within the striatum. This provided an opportunity to compare the operations of feedforward and feedback inhibition.
2. A working memory (WM) of the outcome of pattern classification in positive feedback loops between cortex and thalamus.
3. A recursion-like operation brought about because the loop deposits the WM of prior classifications into an updated input vector from cortex to striatum.

Because of step 3, the updated vector represents prior events, in addition to current events. As a consequence, the next pattern classification step will profit from temporal context. This dependence on past events is a fundamental requirement for serial order processing. Feedback inhibition was more effective and easier to control than feedforward inhibition.

In another modeling study of serial order processing that focused on feedback inhibition in the striatum (Houk et al., 2007), we compared presynaptic inhibition, postsynaptic inhibition and no inhibition to assess their differential performance of a simple serial order task in the presence of noise using a computational model of the cortical-basal ganglionic loop. The best performance was with presynaptic inhibition, next was with postsynaptic inhibition which profited at least from intra-striatal feedback competition, and the worst was with no inhibition.

Imaging studies performed during a serial order recall task additionally provide further evidence for the role of presynaptic inhibition in striatal computation. It has been observed that during the Decode phase of a serial order recall task, normal subjects exhibited a decrease in the Blood Oxygen Level Dependent (BOLD) signal from the caudate nucleus (Houk et al., 2007; See Figure 5 caption for details). No such change was found during Execute relative to rest and an increase was found in the putamen during Execute. The decrease in the caudate during Decode is surprising because neural computation is believed to require elevated levels of synaptic activity, which necessitates an increase in blood flow and translates into an increase in BOLD signal (Logothetis, 2002). Typically, decreases in BOLD are a result of greater synaptic activity during the control task. In the Decode contrast, it could be that the caudate is particularly active during the sensory-guided control task Chase. But it is clear from Figure 5 that the caudate is not significantly active during Execute. Thus, we need another explanation for the significant decrease in BOLD during Decode. One possible explanation is that during Decode presynaptic inhibition is elevated leading to a net decrease in synaptic activity and BOLD signal. Indeed, the model of spiny neuron competition incorporating presynaptic inhibition previously described effectively exhibited decreased BOLD signal in additional to its computational benefits.

**Figure 5:**
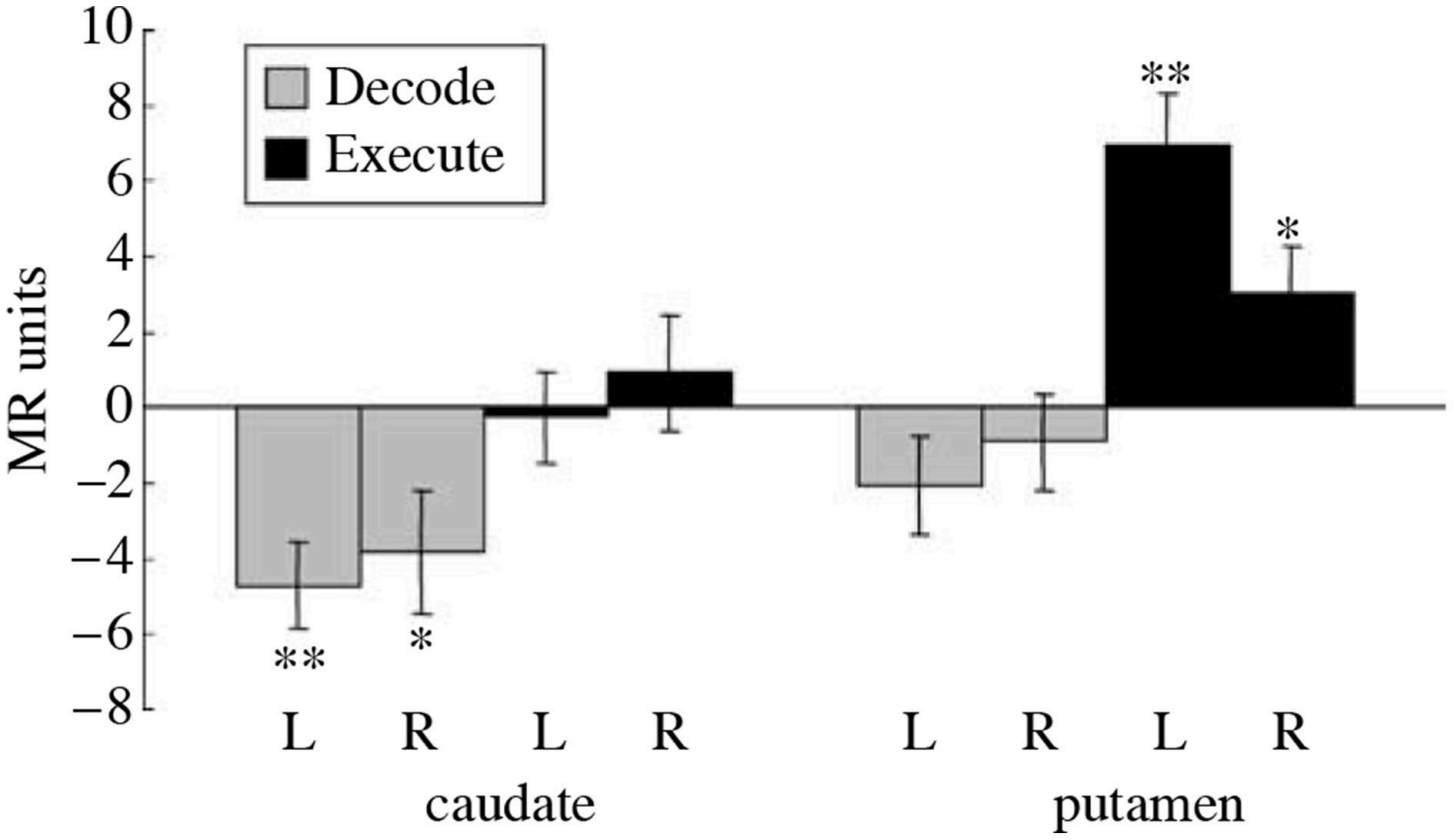
**Reproduced from Houk et al. (2007)**. Differential BOLD activity during a serial order recall task in the right (R) and left (L) caudate and putamen. The entire task, called Replicate, involves the memorization of a pattern of targets, the maintenance of the pattern in working memory, and finally the reproduction of the pattern. Additionally, a control task called Chase was employed where subjects track the target positions immediately, i.e., without a working memory component. The Execute contrast was constructed between the period of sensory-guided target tracking in the Chase task and a rest period, intended to show the neural correlates of motor execution. The Decode contrast was constructed between the memory-guided movement phase of the Replicate task and the sensory-guided movement phase of the Chase task, designed to capture the neural correlates of decoding while controlling for BOLD activity due to pure motor execution. A significant decrease in activity was observed during Decode task in the caudate while a significant increase was observed in the putamen during Execute. The decrease in BOLD during Decode in the caudate implies a decrease in blood flow which is surprising. ^*^indicates a significant difference (*t*_(8)_ ≥ 2.36, *p* < 0.05), while ^**^indicates a highly significant difference.

We have presented the hypothesis that presynaptic inhibition plays a vital functional role in competitive dynamics between spiny neurons in the striatum. Future work should consider the potential role of presynaptic effects explicitly within computational models of competitive inhibition and goal selection in the striatum. Furthermore, ideas presented here may have application toward understanding the origin of schizophrenia, as previously suggested by one of us (Houk, 2012).

Schizophrenia is a complex neuropsychiatric disease with a deficit in WM being a core feature (Lewis and Gonzalez-Burgos, 2006). WM is the process of actively holding information on-line, in the mind's eye, and manipulating it in the service of guiding behavior (Baddeley, 1992). The participation of the dorsolateral prefrontal cortex in WM deficits is now well established, but the nature of the participation has been difficult to understand (Fraser and Houk, 2011). Most helpful has been the study of serial order recall in schizophrenia patients (Fraser et al., 2004) and comparison of the deficits with the Beiser-Houk model of cortical-basal ganglionic processing described earlier. It was concluded that the pattern classification step that goes on in the striatum was operating poorly (Houk, 2012).

A central paradox of schizophrenia is that, in spite of a fecundity disadvantage, a mental illness that is considered to be genetic in origin survives in the population. The magnitude of the deficits are such that any genetic predispositions should be eliminated from the population within a few generations (Berlim et al., 2003). Instead, the incidence of schizophrenia remains steady at about 1%, so one can conclude that there is an accompanying genetic advantage (Crow, 1997). Various authors have suggested three advantageous functions that might accompany the inheritance of a possibility for schizophrenia: (1) a capacity for complex social relations, (2) intelligence, and (3) language (Berlim et al., 2003). Any or all of these advantages are consistent with the prominent disorder in serial order processing in schizophrenia patients that was described previously. This deficit was probably caused by a defect in pattern classification in the striatum of the BG. Pattern classification contributes importantly to the analysis of serial order which is so important in social relations, intelligence and language. Schizophrenia patients perform very poorly when 3, 4, and presumably more items need to be processed in a serial order task (Houk, 2012). While behavior has already been tested, an imaging study could be very rewarding. One could test to see if the decrease in BOLD during the Decode contrast (Figure 5) is absent or reversed, and if it can be manipulated by schizophrenia drugs.

It is worth noting that there are similarities between goal selection in the striatum and future trajectory planning in the hippocampus. In the hippocampus, researchers were unable to connect observed ensemble spiking sequences to the actual decision subsequently made (Johnson and Redish, 2007). It has thus been suggested that these sequences might be representing the space of possible decisions, rather than being involved in the decision making process directly (Wikenheiser and Redish, 2015). Similarly, in the framework of DPMs, the loop through the BG is thought to perform coarse selection of opportune goals that are then refined by the loop through the cerebellum into final decisions, generating time-dependent sequential commands (Rondi-Reig and Burguiere, 2005; Gdowski et al., 2007; Houk, 2012). This perspective thus suggests the disruption of coarse, ballpark goal selection in schizophrenia patients.

## Author contributions

JH and DS both wrote the paper.

## Funding

DJS was partially supported by NIH Grant No. K25 GM098875-02.

### Conflict of interest statement

The authors declare that the research was conducted in the absence of any commercial or financial relationships that could be construed as a potential conflict of interest.
